# Identification of a new electron-transfer relaxation pathway in photoexcited pyrrole dimers

**DOI:** 10.1038/ncomms11357

**Published:** 2016-04-21

**Authors:** Simon P. Neville, Oliver M. Kirkby, Nikolas Kaltsoyannis, Graham A. Worth, Helen H. Fielding

**Affiliations:** 1 School of Chemistry, University of Birmingham, Edgbaston, Birmingham B15 2TT, UK; 2 Department of Chemistry, University College London, 20 Gordon Street, London WC1H 0AJ, UK

## Abstract

Photoinduced electron transfer is central to many biological processes and technological applications, such as the harvesting of solar energy and molecular electronics. The electron donor and acceptor units involved in electron transfer are often held in place by covalent bonds, *π*–*π* interactions or hydrogen bonds. Here, using time-resolved photoelectron spectroscopy and *ab initio* calculations, we reveal the existence of a new, low-energy, photoinduced electron-transfer mechanism in molecules held together by an NH⋯*π* bond. Specifically, we capture the electron-transfer process in a pyrrole dimer, from the excited *π*-system of the donor pyrrole to a Rydberg orbital localized on the N-atom of the acceptor pyrrole, mediated by an N–H stretch on the acceptor molecule. The resulting charge-transfer state is surprisingly long lived and leads to efficient electronic relaxation. We propose that this relaxation pathway plays an important role in biological and technological systems containing the pyrrole building block.

## Abstract (web summary)

Photoinduced electron transfer through, and between, pyrrole moieties is an important process both in the natural world and emerging technologies. Here, the authors use both experiment and theoretical calculation to investigate a previously undiscovered relaxation pathway arising in pyrrole dimers.

## Graphical abstract

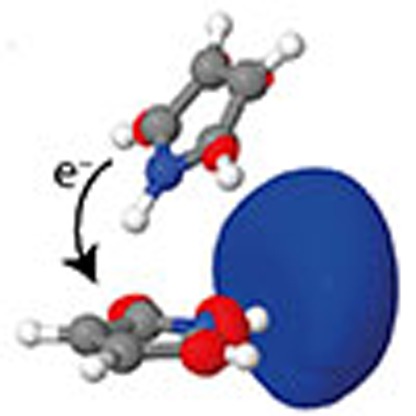

Electron-transfer (ET) reactions in which an electron moves from a donor atom or molecule to an acceptor atom or molecule are ubiquitous in nature and technology^1^,^2^; important examples include the ET chains that drive photosystems I and II (ref. 3) and nanoscale electronic devices^4^. ET processes are often mediated by covalently bound molecular bridges that hold the donor and acceptor in place; for example, in bridged zinc porphyrin-quinone complexes, which are structurally related to the photosynthetic reaction centre, excitation of the porphyrin results in rapid ET to the quinone accompanied by large-scale vibrations of the bridges^5^,^6^. Photoinduced ET can also take place between non-covalently bonded donors and acceptors; for example, between *π*-stacked nucleobases^7^. Recently, there has been a great deal of interest in ET reactions occurring between hydrogen-bonded donors and acceptors accompanied by proton transfer. This class of ET reaction has been shown to provide an efficient mechanism for excited state deactivation following absorption of UV photons. Such proton-coupled ET reactions are important in the photostability of proteins, DNA and other essential bio-macromolecules^8^ and in the photorepair cycles of UV-damaged DNA^9^. Proton-coupled ET has also been found to play an important role in the photostability of hydrogen-bonded heterocycles, such as the pyrrole dimer^10^,^11^.

The pyrrole molecule is a common motif in biology; for example, in tryptophan and porphyrins. It is the key to the photoactivation of the phytochrome enzyme^12^. Pyrrole is also a basic building block in many biologically and technologically important systems, such as polyamide DNA-binding agents in chemical biology^13^,^14^, dye-sensitized solar cells^15^,^16^, the polypyrrole conducting polymer^17^ and self-assembled architectures containing donor–acceptor units^18^. Moreover, it is a prototypical heteroaromatic molecule that undergoes highly efficient non-radiative relaxation on a πσ* potential surface^19^,^20^,^21^. Dimerization of pyrrole has been found to open up additional relaxation pathways, such as a high-energy channel involving proton-coupled ET^10^,^11^.

In this article, we report the observation of a new, low-energy relaxation channel in pyrrole dimers in the gas-phase, involving electron transfer between the two pyrrole molecules. Using time-resolved photoelectron spectroscopy, we find that at high gas pressures, when dimers are present, a spectroscopic feature appears on a sub-picosecond timescale that is not observed at low pressures when only monomers are present. High level *ab initio* calculations show that the lowest excited state of the dimer corresponds to a charge-transfer (CT) state and that the ionization energy of this state is consistent with the spectral feature associated with the dimers. Analysis of the orbitals and molecular structures shows that ET takes place from a *π*-orbital on the donor pyrrole molecule to a Rydberg orbital localized on the N-atom of the acceptor pyrrole molecule. Time-resolved photoelectron spectra recorded following excitation at different wavelengths, together with calculations of potential energy surfaces, suggest that the N–H bond of the acceptor pyrrole must stretch for ET to take place; in the framework of Marcus theory, the N–H bond of the acceptor pyrrole stretches to bring the dimer to a geometry in which there is a strong vibronic coupling between neutral and CT states. As with the high-energy, proton-coupled ET process identified by Slavíček and co-workers^10^,^11^, this new channel competes with dynamics on the πσ* state. A surprising feature of this CT complex is its lifetime. One might assume that, since the dimer is composed of identical units, the back transfer is fast and the CT complex has a negligible lifetime; however, we do not find any evidence for back transfer, which suggests that the CT complex is sufficiently long lived to potentially form a pyrrole ion pair. The elucidation of this new ET mechanism in the pyrrole dimer is significant and may provide a basis for practical applications, such as the rational design of new photoactive materials.

## Results

### Photoelectron spectra of pyrrole and pyrrole clusters

He carrier gas was passed through a reservoir of pyrrole held at 25 °C and the mixture was expanded through a 50 μm diameter, 1-mm-long nozzle. The distribution of pyrrole cluster ions formed by 1+1 ionization of the clusters was 100% monomer when using 0.4 bar He and ∼89% monomer, 10% dimer and 1% trimer when using 1.8 bar He.

**Figure 1:**
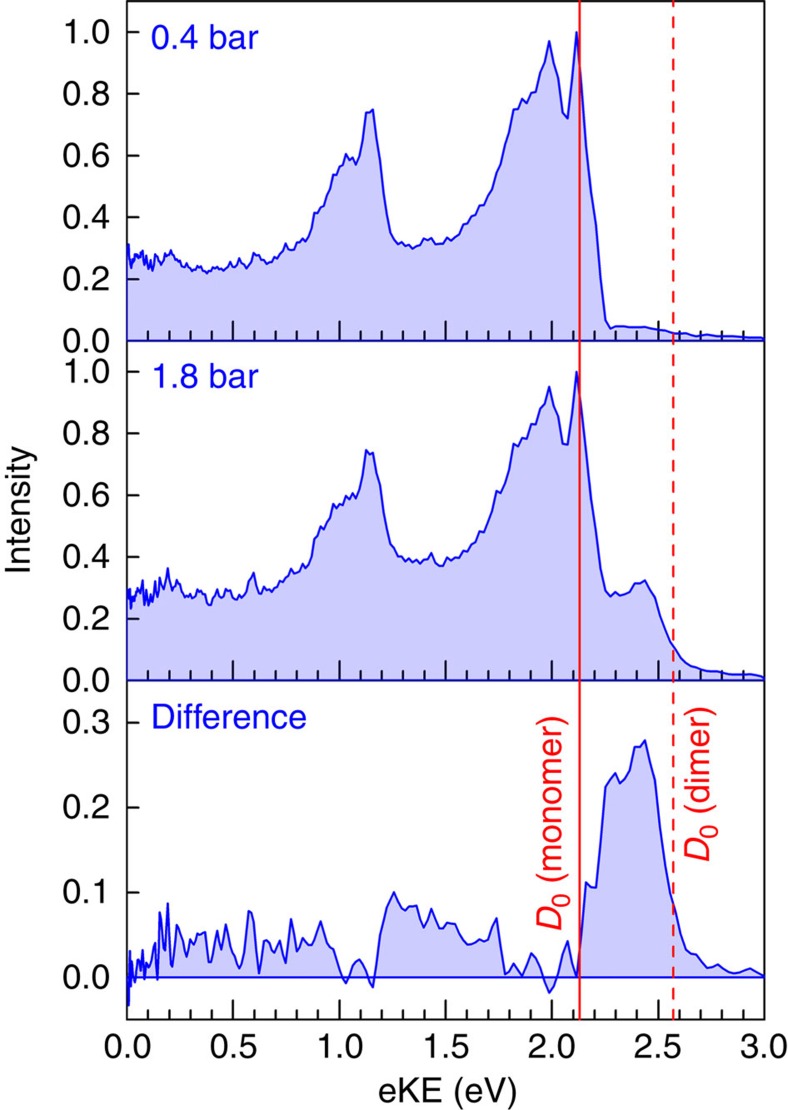
Photoelectron spectra of pyrrole at different backing pressures. 240 nm (5.17 eV) 1+1 photoelectron spectra of pyrrole with He backing pressures of 0.4 bar (top) and 1.8 bar (middle) together with the difference spectrum (bottom) obtained by subtracting the 0.4 bar spectrum from the 1.8 bar spectrum. The 0.4 and 1.8 bar spectra have been normalized to the maximum intensity peak at 2.13 eV. The solid and dashed red lines mark the maximum eKEs possible from 1+1 ionization of the pyrrole monomer and dimer, respectively, calculated using the central wavelength of the laser pulses; the experimental adiabatic ionization potential of the monomer^23^ and the EOM-IP-CCSD/aug-cc-pVDZ calculated vertical ionization energy of the dimer (Table 2).

The spectral signatures of the pyrrole dimer were identifed by comparing photoelectron spectra obtained with different backing pressures of He gas. Figure 1 shows the 240 nm (5.17 eV) 1+1 multiphoton photoelectron spectra, recorded with helium carrier gas pressures of 0.4 and 1.8 bar. At 0.4 bar, the spectrum is identical to the one-photon, vacuum UV photoelectron spectrum of pyrrole^22^, indicating that it is predominantly a 1+1 multiphoton photoelectron spectrum of the monomer. The maximum electron kinetic energy (eKE) possible following 1+1 photoionization of pyrrole, calculated using the central wavelength of the laser pulse and the experimental adiabatic ionization energy (8.207 eV) (refs 23, 24), is 2.13 eV and is marked on Fig. 1. The photoelectron spectrum at 1.8 bar has an additional feature ∼2.45 eV, which lies above the maximum eKE possible following 1+1 photoionization of the monomer. The intensity of this feature is independent of laser power but increases with increasing He backing pressure. At higher pressures, pyrrole clusters are formed and this feature can be attributed to photoionization of these clusters. Since our molecular beam at 1.8 bar is dominated by monomers and the concentration of clusters decreases exponentially with size, it seems most likely that this feature arises from photoionization of pyrrole dimers. To test this suggestion, we have calculated the vertical ionization energies (VIEs) of the monomer, dimer and trimer at the EOM-IP-CCSD/aug-cc-pVDZ level of theory. For the monomer, the lowest VIE is 8.15 eV (ref. 21), in good agreement with the experimental adiabatic ionization energy of 8.207 eV (refs 23, 24). For the dimer, the lowest VIE from the T-shaped minimum energy geometry is 7.77 eV. The pyrrole trimer has three stable conformers and the minimum energy form is predicted by theory and experiment to have a cyclic triangular shape with *C*_3*h*_ symmetry^25^,^26^. From this conformer, the lowest VIE of the trimer is 8.10 eV, very close to that calculated for the monomer. The next most stable trimer structure is 0.16 eV (15 kJ mol^−1^) higher in energy and will not contribute significantly to our experiments. The same trend in VIEs has been calculated at the MP2 level^27^. The optimized geometries for the dimer and trimer at the MP2 level are given in Supplementary Tables 2 and 3.

Using these calculated VIEs, the maximum eKEs for electrons generated by 240 nm 1+1 photoionization of the dimer and trimer are 2.57 and 2.24 eV, respectively, confirming our suggestion that the feature observed around 2.45 eV in the spectrum recorded with 1.8 bar He backing pressure is attributed to 1+1 photoionization of the pyrrole dimer. The 240 nm 1+1 photoelectron spectrum of the dimer can be obtained by subtracting the 0.4 bar spectrum (mostly monomer) from the 1.8 bar spectrum (monomer and clusters) and this is presented in the lower panel of Fig. 1. It should be noted that this is only approximate since the normalization of the two individual spectra is rather arbitrary and there will be some contributions from larger clusters. Nonetheless, it is clear that the photoelectron spectrum of the dimer is dominated by a relatively sharp and symmetric feature centred ∼2.4 eV and that the broad feature that lies under the monomer photoelectron spectrum is significantly weaker than the monomer photoelectron spectrum.

### Electronic structure of the pyrrole dimer

**Figure 2:**
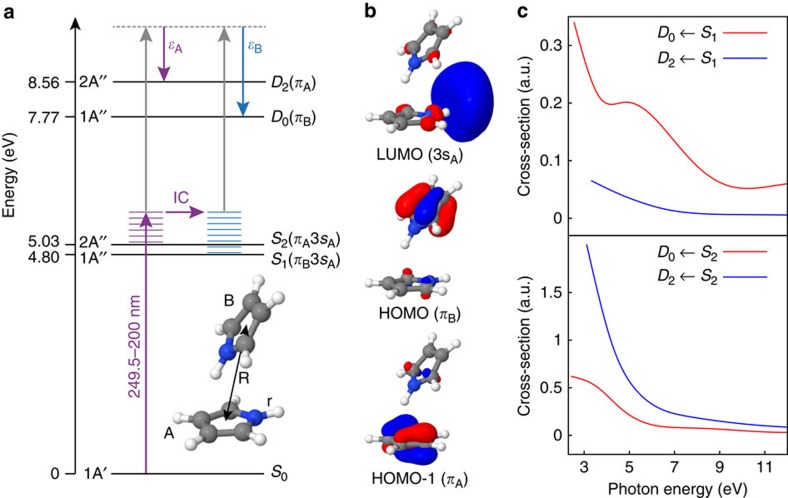
Electronic structure of the pyrrole dimer. (**a**) Energy level diagram showing the ordering of the lowest electronic states of the pyrrole dimer calculated at the CASPT2(8,8) level and the cation calculated at the EOM-IP-CCSD/aug-cc-pVDZ level. Upward arrows represent pump (purple) and probe (grey) laser pulses and the horizontal lines represent vibrational energy in the *S*_1_(*π*_B_3*s*_A_) and *S*_2_(*π*_A_3*s*_A_) states. The horizontal arrow represents IC from the *S*_2_(*π*_A_3*s*_A_) state to the *S*_1_(*π*_B_3*s*_A_) state. Downward arrows represent the eKE of the emitted photoelectrons following *S*_1_(*π*_B_3*s*_A_)→*D*_0_(*π*_B_) and *S*_2_(*π*_A_3*s*_A_)→*D*_2_(*π*_A_) ionization. Inset: ground-state minimum energy geometry of the pyrrole dimer calculated at the SCS-MP2/aug-cc-pVDZ level. The monomer whose N–H bond points into vacuum is labelled A and the other monomer is labelled B. (**b**) Molecular orbitals involved in transitions from *S*_0_ to the *S*_1_(*π*_B_3*s*_A_) and *S*_2_(*π*_A_3*s*_A_) states of the neutral dimer. (**c**) Photoionization cross-sections, as a function of photon energy, for ionization from the *S*_1_(*π*_B_3*s*_A_) and *S*_2_(*π*_A_3*s*_A_) states to the *D*_0_(*π*_B_) and *D*_2_(*π*_A_) cation states, calculated at the EOM-IP-CCSD/aug-cc-pVDZ level. IC, internal conversion.

The pyrrole dimer is known to have a T-shaped structure with *C*_s_ symmetry^25^,^26^, in which the two pyrrole molecules are held together by an NH⋯*π* bond (Fig. 2a), a motif found in protein structures^28^,^29^. This non-typical hydrogen bond is also responsible for the self-assembly of pyrrole dimers in apolar solvents^30^. The monomer whose N–H bond points into vacuum is labelled A and the monomer whose N–H bond is directed towards the *π* electron system of A is labelled B. The centres of mass of the monomers are calculated to be separated by *R*=4.06 Å, the planes of the monomers are at angle of *φ*=51.5°, and the centre of mass vector has an angle of *θ*=13.6° from the norm of the plane of monomer A, in agreement with the results of gas-phase experiments^31^,^32^ and other *ab initio* calculations^11^,^33^, including recent dispersion-corrected density functional theory (DFT) calculations^34^. The latter study also employed molecular dynamics simulations to show that the NH⋯*π* bond persists in liquid pyrrole.

The results of electronic structure calculations for the pyrrole dimer are summarized in Tables 1 and 2 and the energy level diagram in Fig. 2a. At all levels of theory, both the *S*_1_ and *S*_2_ states of the neutral dimer are found to be of A′′ symmetry and have 3*s* character at the Franck–Condon point. The *S*_1_(*π*_B_3*s*_A_) state has CT character, with the dominant configuration corresponding to excitation from a *π*-orbital localized on monomer B to the 3*s* orbital localized on the N-atom of monomer A (Fig. 2b). In contrast, the *S*_2_(*π*_A_3*s*_A_) state is dominated by a configuration corresponding to excitation from a *π*-orbital localized on monomer A to the 3*s* orbital localized on the same monomer (Fig. 2b), reminiscent of the A_2_(*π*3*s*) state of the pyrrole monomer^35^.

The *D*_1_(*π*_A_) and *D*_2_(*π*_A_) states of the pyrrole dimer cation have A′ and A′′ symmetries, respectively, and are dominated by configurations corresponding to removal of an electron from *π*-orbitals localized on monomer A. From a consideration of the dominant configurations and the symmetries of these states, the dimer cation states *D*_1_(*π*_A_) and *D*_2_(*π*_A_) correlate with the *D*_1_(*π*) and *D*_0_(*π*) states of the monomer cation, which have symmetries B_1_ and A_2_ respectively, and correspond to removing an electron from the HOMO-1 *π*-orbitals. The *D*_0_(*π*_B_) dimer cation state is dominated by a configuration corresponding to removal of an electron from a *π*-orbital localized on monomer B and lies below the lowest two monomer-like states with a calculated VIE of 7.77 eV.

The photoionization cross-sections corresponding to ionization to the *D*_0_(*π*_B_) and *D*_2_(*π*_A_) cation states from the *S*_1_(*π*_B_3*s*_A_) and *S*_2_(*π*_A_3*s*_A_) dimer neutral states are plotted in Fig. 2c. The *S*_1_(*π*_B_3*s*_A_) state is predicted to ionize preferentially to the *D*_0_(*π*_B_) state in the region of the probe photon energy (4.13 eV), while the *S*_2_(*π*_A_3*s*_A_) state is predicted to ionize preferentially to the higher lying *D*_2_(*π*_A_) state. The cross-section for ionization to the *D*_1_(*π*_A_) state of the cation is close to zero for ionization from either neutral excited state, similar to the negligible photoionization cross-section found for the *S*_1_(*π*3*s*) state of the pyrrole monomer to the monomer cation *D*_1_(*π*) state^21^.

### Time-resolved photoelectron spectra

**Figure 3:**
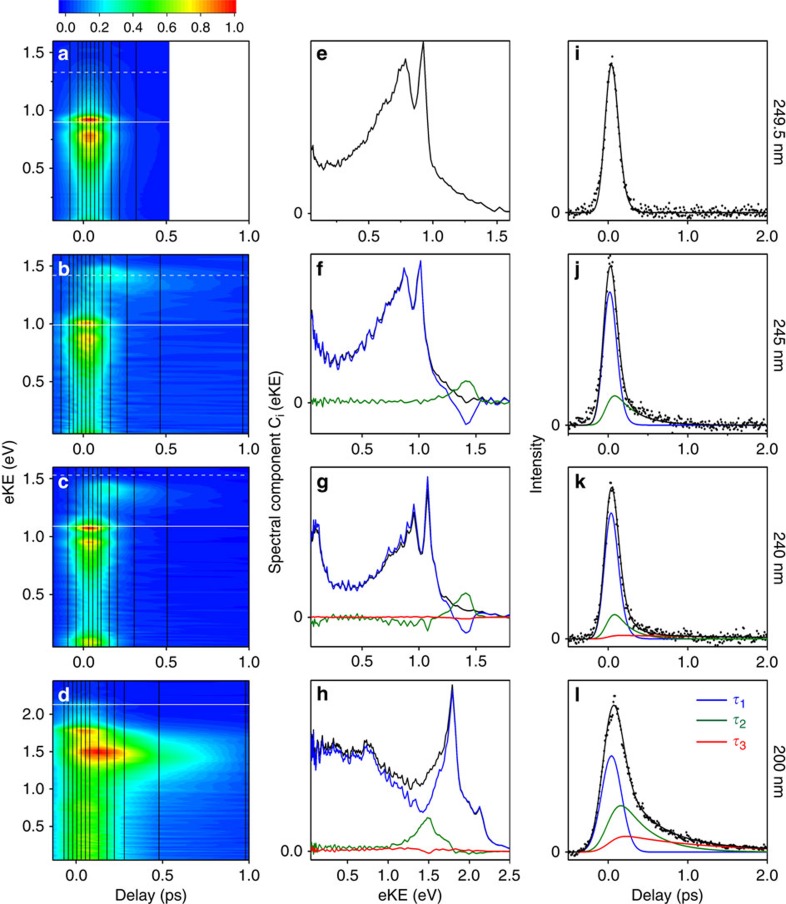
Excited state dynamics of pyrrole at 1.8 bar. (**a**–**d**) Contour plots showing experimental time-resolved photoelectron spectra following excitation at 249.5 nm (4.97 eV), 245 nm (5.06 eV), 240 nm (5.17 eV) and 200 nm (6.20 eV). Individual plots were normalized to their maximum photoelectron signals. The vertical black lines mark the pump–probe delays at which photoelectron spectra were recorded and the shading was smoothed using linear interpolation. The horizontal white lines mark the maximum eKEs possible from 1+1′ ionization, calculated using the central wavelengths of the pump and probe laser pulses and the experimental adiabatic ionization potentials of the pyrrole monomer, 8.207 eV (solid lines)^23^, and EOM-IP-CCSD/aug-cc-pVDZ calculated vertical ionization energies of the pyrrole dimer (dashed lines), 7.77 eV (this work). (**e**–**h**) Spectral components of *C*_*i*_(e*KE*) extracted from the time-resolved photoelectron spectra using the decay times listed in Table 3 and equation (1). The sum of *C*_*i*_(e*KE*) (black lines) represents the combined photoelectron spectrum of the initially excited states of the pyrrole monomer and dimer. (**i**–**l**) Integrated photoelectron spectra of the initially populated momomer and *S*_2_(*π*_A_3*s*_A_) state of the dimer (blue) that decay on a timescale <60 fs, the *S*_1_(*π*_B_3*s*_A_) state of the dimer (green) whose population rises on a timescale <60 fs and subsequently decays on a timescale 190–360 fs, the longer-lived excited state population (red) and the total integrated photoelectron spectrum (black), together with experimental data (scattered points).

Figure 3a–d shows the time-resolved 1+1′ photoelectron spectra recorded using 1.8 bar He carrier gas (monomers and clusters) with pump wavelengths of 249.5 nm (4.97 eV), 245 nm (5.06 eV), 240 nm (5.17 eV) and 200 nm (6.20 eV) and a probe wavelength of 300 nm (4.13 eV). It is worth noting that the eKE axes are not directly comparable with those in Fig. 1 because different wavelengths are employed in the 1+1 and 1+1′ photoionization schemes.

For each photoexcitation wavelength, the integrated areas of the photoelectron spectra have been scaled to the total integrated photoelectron signals at the corresponding pump–probe delays and plotted as a contour map. No obvious variation in anisotropy parameter with pump–probe delay was observed. The lifetimes determined from the time-resolved photoelectron spectra are presented in Table 3. To gain insight into the flow of excited-state population, the time-resolved photoelectron spectra were fit to sums of the exponential decays listed in Table 3 and convoluted with the pump–probe cross-correlation *g*(*t*),


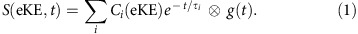


The coefficients *C*_*i*_(eKE) represent the contribution of the *i*th decay at a given eKE. The spectra of the fit coefficients *C*_*i*_(eKE) are plotted in Fig. 3e–h. Positive values of *C*_*i*_(eKE) represent exponential decay on a timescale *τ*_*i*_ and negative values represent exponential growth on a timescale *τ*_*i*_.

Only a single lifetime, *τ*_1_=47 fs, was required to fit the 249.5 nm data. The spectrum associated with this lifetime (Fig. 3e) has positive amplitudes everywhere, indicating a rapid decay of population out of the photoionization window. Following excitation at 245 and 240 nm, the spectra associated with the *τ*_1_ timescale have positive amplitudes in the range 0–1.3 eV but negative amplitudes ∼1.45 eV (Fig. 3f,g). This suggests an evolution along the excited potential energy surface from a region with a photoelectron spectrum with lower eKE to a region with a photoelectron spectrum with higher eKE. At 245 and 240 nm, spectra associated with a second ultrafast timescale, *τ*_2_≈190–270 fs, are centred around 1.45 eV with positive amplitude, suggesting that once populated, the region of the excited potential energy surface with a photoelectron spectrum with higher eKE decays out of the photoionization window with a slightly longer timescale *τ*_2_. This sequential population and decay is clearly visible as a delayed rise and fall of photoelectron signal ∼1.45 eV in Fig. 3b,c as well as in plots showing the integrated photoelectron signals corresponding to *τ*_1_ and *τ*_2_ (Fig. 3j,k). At 240 nm, a third lifetime is required to fit the data, *τ*_3_≈2.5 ps. The spectrum associated with this timescale has very low amplitude, which is either the signature of larger clusters or may be attributed to a small fraction of the population remains trapped on the excited potential energy surface of the dimer within the photoionization window and decaying on a much slower timescale. At 200 nm, the situation is similar to that for 240 nm. The spectrum associated with the *τ*_1_ timescale has positive amplitudes across the range 0–2.5 eV, but there is a dip in the amplitude ∼1.45 eV that coincides with the spectrum associated with the *τ*_2_ timescale that has positive amplitude. This is consistent with a flow of population from a region of the excited potential energy surface that has a broad photoelectron spectrum with maximum eKE ∼2.5 eV to a region of the photoelectron spectrum that has a maximum eKE ∼1.45 eV. A third lifetime, *τ*_3_≈1.1 ps, is also required to fit the 200 nm photoelectron spectra. Again, the spectrum associated with this timescale has low amplitude; however, it is larger than the equivalent spectrum for 240 nm and has a small dip ∼1.5 eV. Again, this is either a signature of larger clusters or it may be attributed to population that remains trapped on the excited potential energy surface of the dimer and flows into the region of the photoelectron spectrum with maximum eKE ∼1.45 eV.

## Discussion

The time-resolved photoelectron spectra are dominated by the excited-state dynamics of the pyrrole monomer. Recently, Wu *et al*. used time-resolved photoelectron spectroscopy to probe the relaxation dynamics of the pyrrole monomer following excitation in the range 242–217 nm (5.1–5.7 eV) (ref. 21). They found that photoexcitation in this range resulted in ultrafast decay of the system from the ionization window on a timescale <30 fs, in agreement with other femtosecond studies^36^,^37^,^38^,^39^. Our time-resolved photoelectron spectra of the pyrrole monomer using 0.4 bar He carrier gas and the lifetimes (Supplementary Table 1) are in agreement with those of Wu *et al*. The main difference between the time-resolved photoelectron spectra recorded at 0.4 and 1.8 bar is the feature around 1.45 eV, which has already been assigned to the dimer and is observed to have a delayed rise on an ultrafast timescale (<60 fs) and subsequent decay on a slightly longer timescale (190–360 fs).

To a first approximation, the photoelectron spectrum of the dimer can be understood using conservation of energy. Assuming the excess vibrational energy for a given electronic state, *E*_vib_=*hν*_pump_−*E*(*S*_*n*_), is conserved during photoionization, eKE≈*hν*_probe_−[*E*(*D*_*n*_)−*E*(*S*_*n*_)]. Thus, from the vertical excitation energies (VEEs) of the neutral dimer calculated at the CASPT2 level (Table 1) and the VIEs (Table 2), ionization from the *S*_2_(*π*_A_3*s*_A_) state of the dimer to the *D*_2_(*π*_A_) state is predicted to produce photoelectrons with eKE∼0.6 eV, coinciding with the photoelectron spectrum of the monomer. The *S*_1_(*π*_B_3*s*_A_) state is predicted to ionize preferentially to the *D*_0_(*π*_B_) cation and is predicted to produce photoelectrons with eKE∼1.2 eV. Thus, our photoelectron spectra show that the *S*_2_(*π*_A_3*s*_A_) state of the dimer, which has a similar photoelectron spectrum to the *S*_1_(*π*3*s*) state of the monomer, is populated directly and has a lifetime that is indistinguishable from that of the *S*_1_(*π*3*s*) state of the monomer. The *S*_1_(*π*_B_3*s*_A_) CT state of the dimer is populated indirectly, from the *S*_2_(*π*_A_3*s*_A_) state on a timescale of <60 fs and subsequently decays on a slightly longer timescale (190–360 fs).

**Figure 4:**
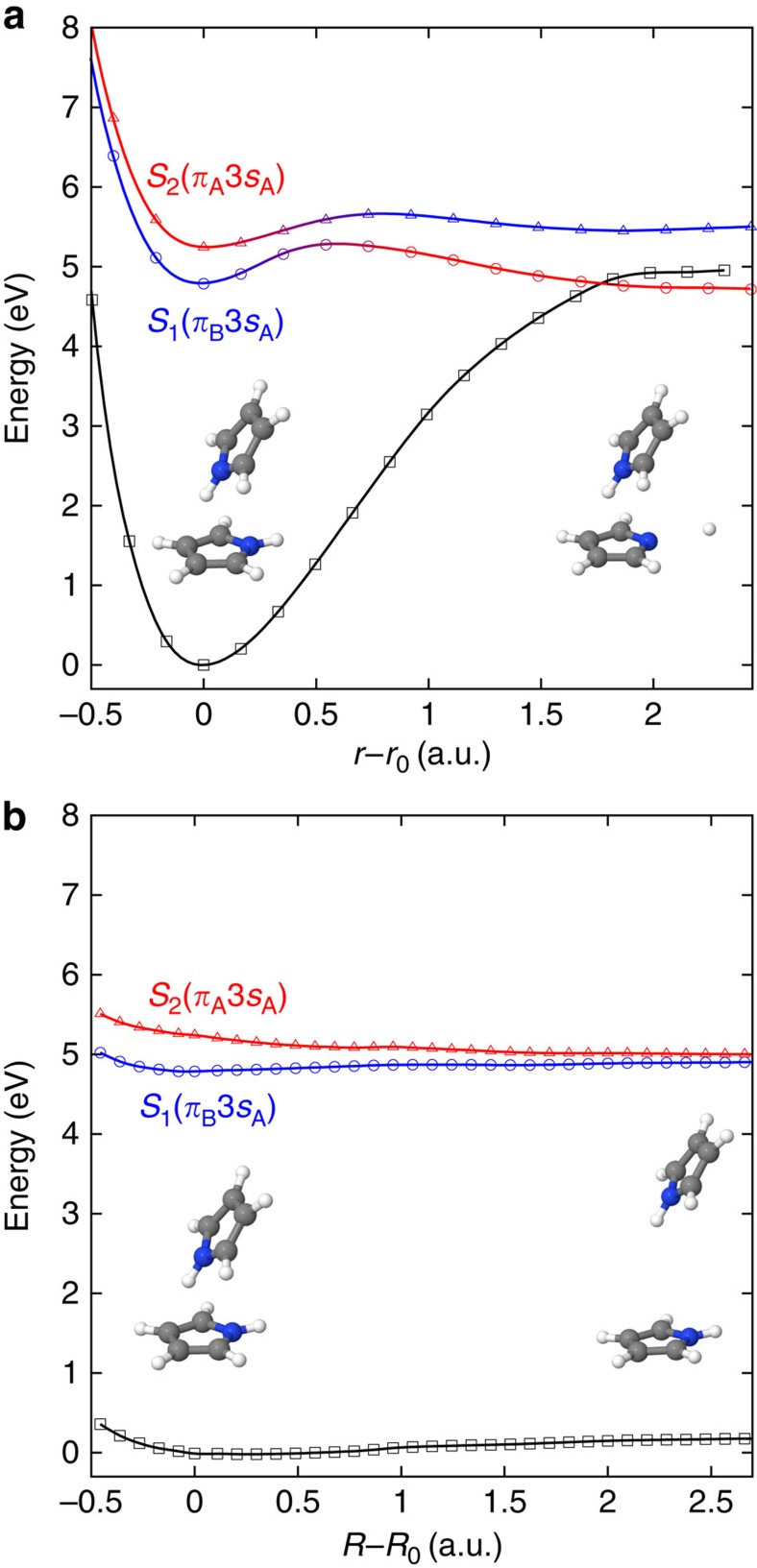
Electron-transfer process between non-covalently bonded pyrrole molecules. Adiabatic potential energy curves for the *S*_0_ (black), *S*_1_(*π*_B_3*s*_A_) (blue) and *S*_2_(*π*_A_3*s*_A_) (red) states calculated at the DFT/MRCI/aug-cc-pVDZ level, (**a**) along the N–H dissociation coordinate of monomer A and (**b**) along the vector ***R*** connecting the centres of masses of the two monomers.

For the dimer, the calculated VEEs (Table 1), confirm that excitation at 249.5, 245 and 240 nm will result predominantly in population of the *S*_2_(*π*_A_3*s*_A_) state, which has an oscillator strength an order of magnitude higher than the *S*_1_(*π*_B_3*s*_A_) state (Supplementary Table 4). Excitation at 200 nm is likely to result in transitions to higher lying optically bright *π*→π* or *π*→3*p* states, predominantly localized on monomer A (Supplementary Table 4). The potential energy surfaces of the *S*_0_, *S*_1_(*π*_B_3*s*_A_) and *S*_2_(*π*_A_3*s*_A_) states along the N–H dissociation coordinate ***r*** of monomer A and the vector ***R*** connecting the centres of mass of the two monomers are presented in Fig. 4. In the N–H dissociation limit, the adiabatic *S*_1_ state correlates with the diabatic *S*_2_(*π*_A_3*s*_A_) state, while the adiabatic *S*_2_ state correlates with the diabatic *S*_1_(*π*_B_3*s*_A_)state. This change in character is represented on the plot by a change in colour on the adiabatic curves along the N–H stretching coordinate ***r***. In a diabatic picture, the *S*_1_(*π*_B_3*s*_A_) CT state is essentially bound with respect to N–H dissociation and the *S*_2_(*π*_A_3*s*_A_) state has only a shallow barrier to dissociation. However, in the adiabatic picture, the N–H bond becomes bound in the *S*_2_(*π*_A_3*s*_A_) state due to the avoided crossing with the lower *S*_1_(*π*_B_3*s*_A_) CT state. The *S*_1_(*π*_B_3*s*_A_) state is found to be weakly bound with respect to the monomerization coordinate ***R***, with a barrier to monomerization of around 0.1 eV, whereas the *S*_2_(*π*_A_3*s*_A_) state potential is found to be repulsive along this ***R*** coordinate. Motions along the ***r*** and ***R*** coordinates will be the key initial motions because the N–H bond in pyrrole is photolabile and the cluster is only weakly bound; however, motions along other degrees of freedom may also be involved in the electronic relaxation pathway. For example, motion along the N out-of-plane coordinate and some ring stretches are known be involved in the relaxation dynamics of pyrrole^35^ so may also play a role in the relaxation of the pyrrole dimer. Motion along a tilting coordinate that brings the monomers into co-planarity may play a role at longer timescales since it is known that in the benzene dimer the monomers are co-planar^40^ and such a geometry might stabilize the CT state in the pyrrole dimer. Nonetheless, these motions will not change the major character of the initial dynamics which will be dominated by motions along the N–H stretch and monomerization coordinates.

At 249.5 nm, a single ultrafast decay out of the photoionization window of the predominantly populated *S*_2_(*π*_A_3*s*_A_) state is observed. Since there is only one timescale, which is indistinguishable from that of the monomer, it seems that monomer separation together with ultrafast N–H dissociation of monomer A is likely to be the predominant decay channel of the dimer at this excitation energy. At this wavelength, excitation is to the bottom of the *S*_2_(*π*_A_3*s*_A_) potential well plotted as a function of the N–H bond length; however, as soon as the dimers start to separate along the dissociative *R* monomerization coordinate, the barrier to N–H dissociation will approach that of the monomer and thus the dimer will display only monomer-like behaviour.

At 245 and 240 nm, ultrafast decay out of the photoionization window is still observed, but there is a delayed rise and subsequent decay of the higher-energy dimer band around 1.45 eV (Fig. 3b,c), which is consistent with a competing process populating the *S*_1_(*π*_B_3*s*_A_) state. At 200 nm, a similar ultrafast population and subsequent decay of the *S*_1_(*π*_B_3*s*_A_) state is observed, also ∼1.45 eV because of the propensity for conservation of excess vibrational energy during photoionization. These observations suggest that a crossing point between the *S*_1_(*π*_B_3*s*_A_) and *S*_2_(*π*_A_3*s*_A_) states becomes accessible between 249.5–245 nm (4.97–5.06 eV) and crossing occurs to form the CT complex on a timescale that is faster than that of monomer separation in the *S*_2_(*π*_A_3*s*_A_) state. This population is likely to occur at the avoided crossing between the *S*_1_(*π*_B_3*s*_A_) and *S*_2_(*π*_A_3*s*_A_) states along the N–H bond stretch (Fig. 4). However, as discussed above, motions along other coordinates are also expected to play a role in the dynamics, possibly turning this avoided crossing into a conical intersection (although as the states have the same symmetry this would be an accidental intersection) and moving towards co-planarity. Nonetheless, these motions will not change the basic picture of initial N–H stretching followed by crossing to form the CT state that must occur.

It can also be inferred that the decay time *τ*_2_∼190–360 fs (Table 3) is the lifetime of the dimer CT complex. However, how the CT complex relaxes cannot be ascertained from our experiments and calculations and various pathways are possible. Significantly, there is no experimental evidence for re-crossing from the CT complex back to the neutral dimer excited state. This is surprising given the equivalent electronegativities of the monomers. Another possibility might be N–H dissociation in the *S*_1_(*π*_B_3*s*_A_) state, but it should be noted that the barrier to H-atom loss in the *S*_1_(*π*_B_3*s*_A_) state (0.5 eV, calculated at the DFT/multi-reference configuration interaction (MRCI) level), is significantly larger than that of the monomer (0.25 eV (ref. 35)). The most plausible pathways are: direct crossing to the ground-state, as suggested by Poterya *et al*.^10^; slow separation to form a monomer pair as the barrier to monomerization calculated to exist in the *S*_1_(*π*_B_3*s*_A_) state at the DFT/MRCI level is very small (Fig. 4); or formation of a co-planar excimer ion pair as seen in benzene^40^ if the tilt motion becomes important during separation.

In conclusion, the dynamics of the excited states of the pyrrole dimer have been studied using a combination of time-resolved photoelectron spectroscopy and *ab initio* calculations. It has been revealed that the formation of pyrrole dimers opens up a new relaxation pathway involving ET from the excited *π*-system of one pyrrole molecule to a Rydberg orbital localized on the N-atom of the other pyrrole molecule and that the ET is mediated by stretching of the N–H bond on the acceptor molecule leading to efficient curve crossing. Furthermore, our results reveal that the resulting CT complex has a lifetime of a few 100 fs and that it may form a pyrrole ion pair. We propose that this relaxation pathway will play a role in the photochemistry and photophysics of biologically and technologically important systems containing the pyrrole building block or indeed other NH–*π* bonded molecular units.

## Methods

### Cluster source

Helium carrier gas (0.4–1.8 bar) was passed through a reservoir of liquid pyrrole held at room temperature (25 °C) outside the vacuum chamber and carried into the vacuum chamber through 1/16′′ tubing to a 1-mm-long nozzle of 50 μm diameter. The vapour pressure of pyrrole at 25 °C is 8.35 mm Hg and the concentration of pyrrole in 1.8 bar He is estimated to be 0.6%. The molecular beam passed through a 1 mm skimmer, located ∼40 mm from the nozzle, into the interaction region of a photoelectron velocity map imaging spectrometer^41^,^42^,^43^. To confirm the formation of clusters, we recorded ion images following two-photon ionization at 249.5 nm. The time-of-flight from the interaction region to the detector depends on the mass of the ion, 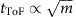, and we observe images of dimer ions displaced by 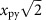 and trimer ions displaced by 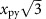, where *x*_py_ is the displacement along the molecular beam axis of the pyrrole monomer ions, which is perpendicular to the time-of-flight axis. With a carrier gas pressure of 0.4 bar, 100% of the ion signal corresponded to *m*/*z*≡C_4_H_5_N^+^. At 1.8 bar, the cluster ion distribution at the detector is exponential with around 89% monomer, 10% dimer and 1% trimer (Supplementary Fig. 1 and Supplementary Note 1) and the average cluster ion size at the detector is 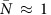. We note that these measurements are lower limits for the fractions of clusters in the interaction region because they do not account for fragmentation of cluster ions on the way to the detector. Nonetheless, our observations are consistent with those of Poterya *et al*.^10^ and Profant *et al*.^27^ who measured average cluster sizes 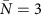 following supersonic expansion of pyrrole through a 2-mm-long conical nozzle of 60 μm diameter, with a He backing pressure of 1.5 bar and pyrrole reservoir and nozzle temperatures 8 and 9 °C, respectively, substantially lower than those in our experiments.

### Time-resolved photoelectron spectra

The time-resolved photoelectron spectra were recorded with helium gas pressures of 1.8 bar. After collimation by a 1 mm skimmer, the molecular beam was intersected by femtosecond pump (249.5–200 nm) and probe (300 nm) laser pulses. Pump–probe cross-correlation full-width half-maximum measurements were in the range 180–290 fs. The pump and probe pulses were focussed to a spot size of diameter ∼50 μm after being attenuated to <1 μJ per pulse (<10^11^ W cm^2^) to minimize multiphoton processes and space-charge effects. For each excitation wavelength, a set of ∼15 photoelectron images with pump–probe delays in the range −0.25 to 1 ps were recorded, together with the total integrated photoelectron signal. Photoelectron velocity distributions were recovered from the raw photoelectron images using the pBasex image inversion algorithm^44^ and the energy scale was calibrated by recording the 2+1 resonance-enhanced multiphoton ionization spectrum of Xe at 249.6 nm (ref. 45). The resolution of this instrument is ∼3.5%. To extract decay times from a set of time-resolved photoelectron spectra, the total integrated areas of the photoelectron spectra recorded at each pump–probe delay were scaled to the total integrated photoelectron signal intensity. A least-squares fitting procedure using the Levenberg–Marquardt optimization algorithm was used to fit integrated portions of the set of scaled spectra to sums of exponentially decaying profiles convoluted with a Gaussian cross-correlation function, representing the cross-correlation of the pump and probe laser pulses *g*(*t*) (refs 43, 46),


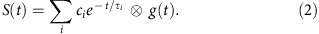


*c*_*i*_ is the intensity of the *i*th decay with 1/*e* decay time *τ*_*i*_. The residuals from the fits are shown in Supplementary Fig. 2.

### Computational methodology

Ground-state minimum energy geometries of the pyrrole monomer, dimer and trimer were optimized at the spin-component-scaled MP2 (SCS-MP2)^47^ level using the aug-cc-pVDZ basis. Ionization potentials for the lowest cationic states were calculated at the EOM-IP-CCSD/aug-cc-pVDZ level. For the dimer, vertical excitation energies of the low-lying excited states at the Franck–Condon point geometry were calculated using the EOM-CCSD, CASSCF, CASPT2 and DFT/MRCI methods and the aug-cc-pVDZ basis set. The DFT/MRCI calculations^48^ were performed using the BH-LYP functional. The CASSCF and CASPT2 calculations were carried out using eight electrons in eight orbitals and the aug-cc-pVDZ basis set, that is, CAS(8,8)/aug-cc-pVDZ and CASPT2(8,8)/aug-cc-pVDZ. The active space was the four occupied orbitals 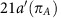, 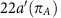, 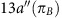 and 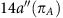, and four virtual orbitals 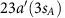, 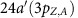, 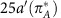 and 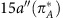. The subscripts *A* and *B* denote the monomer on which a given orbital is localized. The CAS calculations were performed using MOLPRO^49^, the EOM-CCSD calculations using QChem^50^ and the MP2 and DFT/MRCI calculations using Turbomole^51^.

Photoionization cross-sections, 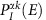, corresponding to ionization from the low-lying excited neutral states were calculated under the assumption of vertical excitation and the applicability of Fermi's golden rule,


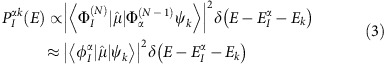


Here, the 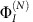 and 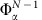 correspond to the *N*-electron neutral and (*N*-1)-electron cationic states, respectively, while *ψ*_*k*_ denotes the photoelectron orbital (taken as a Coulomb partial wave with kinetic energy *E*_*k*_=*k*^2^/2). The energy of the applied laser pulse is denoted by *E*, whilst 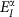 denotes the vertical energy difference between the netural and cationic states, and 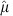 is the molecular dipole operator. The one-electron quantity 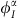 is a so-called Dyson orbital, obtained from the overlap of the neutral and cationic states^52^.

Dyson orbitals corresponding to ionization from both the *S*_1_ and *S*_2_ states to each of the *D*_0_, *D*_1_ and *D*_2_ states were calculated at the EOM-CCSD/EOM-IP-CCSD level using the aug-cc-pVDZ basis set. Evaluation of the photoionization matrix elements 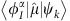 was performed numerically on a grid and with isotropic averaging over molecular orientations using the ezDyson program^53^.

## Additional information

**How to cite this article:** Neville, S. P. *et al*. Identification of a new electron-transfer relaxation pathway in photoexcited pyrrole dimers. *Nat. Commun.* 7:11357 doi: 10.1038/ncomms11357 (2016).

## Supplementary Material

Supplementary Information Supplementary Figures 1-2, Supplementary Tables 1-4, Supplementary Note 1, Supplementary Methods and Supplementary References.

## Figures and Tables

**Table 1:** VEEs of the pyrrole dimer.

**State**	**Configuration**	**Δ*****E*****(CASSCF)**	**Δ*****E*****(CASPT2)**	**Δ*****E*****(EOM-CCSD)**	**Δ*****E*****(DFT/MRCI)**
*S*_1_, 1A′′	*π*_B_→3*s*_A_	4.84	4.80	5.19	4.79
*S*_2_, 2A′′	*π*_A_→3*s*_A_	5.18	5.03	5.56	5.24

DFT, density functional theory; MRCI, multi-reference configuration interaction; VEE, vertical excitation energy.

VEEs and dominant configurations of the first two excited states of the pyrrole dimer calculated at the CASSCF(8,8), CASPT2(8,8), EOM-CCSD and DFT/MRCI levels using the aug-cc-pVDZ basis. VEEs are given in units of eV.

**Table 2:** VIEs of the pyrrole dimer.

**State**	**Configuration**	**VIE**
*D*_0_, 1A′′	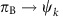	7.77
*D*_1_, 1A′′	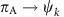	8.52
*D*_2_, 2A′′	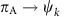	8.56

VIE, vertical ionization energy.

VIEs and dominant configurations of the first three cationic states of the pyrrole dimer, calculated at the EOM-IP-CCSD level using the aug-cc-pVDZ basis set. VIEs are given in units of eV.

**Table 3:** Summary of pyrrole lifetimes and pump–probe cross-correlation measurements.

***λ***_**pump**_**/nm**	***τ***_**1**_**/fs**	***τ***_**2**_**/fs**	***τ***_**3**_**/ps**	***g*****(*****t*****)/fs**
249.5	47±1			180±1
245	46±8	270±40		179±8
240	57±8	190±30	2.5±1.5	179±9
200	31±1	360±120	1.1±0.2	289±3

The 1/*e* lifetimes and pump–probe cross-correlation, *g*(*t*), extracted from time-resolved photoelectron spectra of pyrrole using 1.8 bar He carrier gas with different pump wavelengths. The errors quoted represent 2 s.d.
