# Supplementary material for: Identification of a new electron-transfer relaxation pathway in photoexcited pyrrole dimers
Source: Nat Commun. 2016 Apr 21;7:11357. doi: 10.1038/ncomms11357 (PMC4844682; doi:10.1038/ncomms11357)
Supplement: Supplementary Information — Supplementary Figures 1-2, Supplementary Tables 1-4, Supplementary Note 1, Supplementary Methods and Supplementary References. [file ncomms11357-s1.pdf]

Supplementary Figures

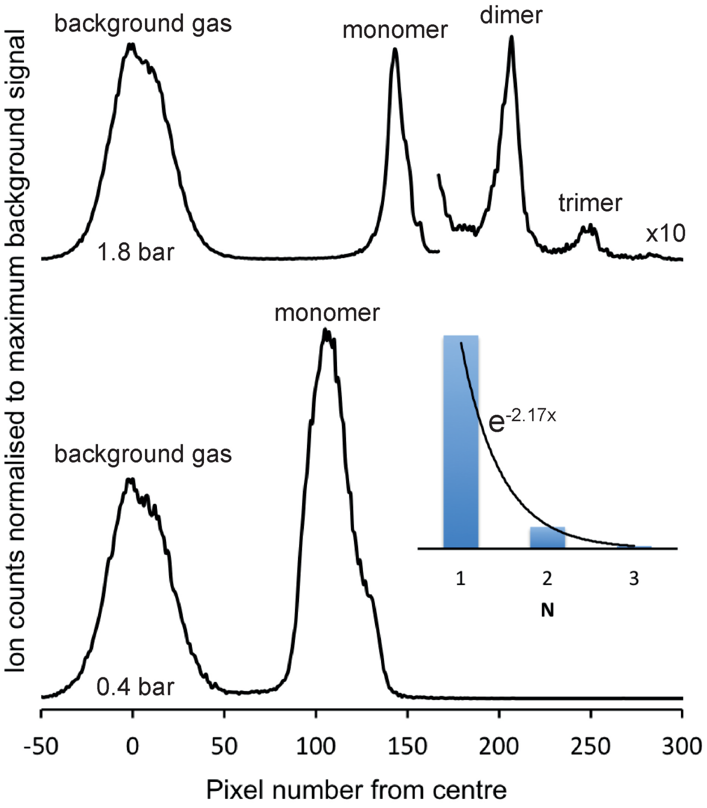

Supplementary Figure 1: Integrated ion counts as a function of displacement along the direction of propagation of the molecular beam, from the laser interaction region, with He backing pressures of 0.4 bar (lower trace) and 1.8 bar (upper trace); the two plots are normalised to the maximum background gas counts. The inset is a plot of the relative integrated ion counts for pyrrole clusters of size *N*, together with a least squares fitted exponential decay.

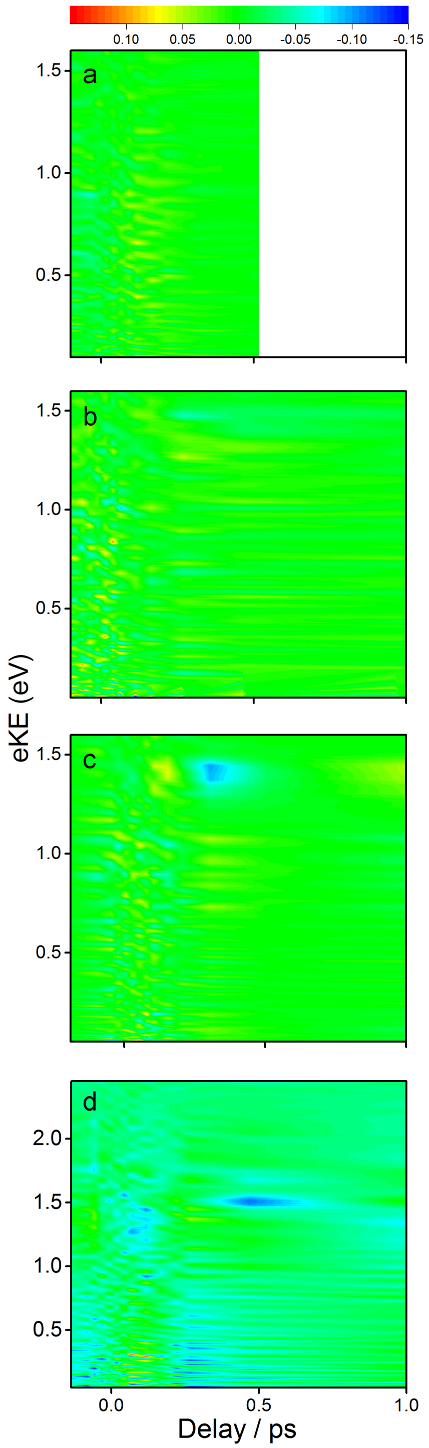

Supplementary Figure 2: Residuals of the photoelectron spectra fitted to eq. (1) in the paper subtracted from the experimental photoelectron spectra recorded following excitation at (a) 249.5 nm, (b) 245 nm, (c) 240 nm and (d) 200 nm. The intensity scales are relative to the normalised intensities of the photoelectron spectra and the shading was smoothed using linear interpolation.

Supplementary Tables

λ_{pump}/nm	$g(t)$/fs	τ_1/fs
249.5	187 ± 2	39 ± 3
245	175 ± 1	49 ± 1
240	204 ± 1	22 ± 1
200	308 ± 2	29 ± 4, 35 ± 2

Supplementary Table 1: $1/e$ lifetimes and pump-probe cross-correlation measurements, $g(t)$, extracted from time-resolved photoelectron spectra of the pyrrole monomer recorded with 0.4 bar He pressure. The errors quoted represent two standard deviations of the fit. The error in the measurement is likely to be around 10 fs. The values reported here are in agreement with the lifetimes measured by Wu *et al.* over a similar range of excitation wavelengths[1].

C	0.472476	1.921374	1.13339
C	-0.797600	2.331123	0.71858
C	-0.797600	2.331123	-0.71858
C	0.472476	1.921374	-1.13339
H	0.891223	1.779573	2.12785
H	-1.621265	2.610388	1.37507
H	-1.621265	2.610388	-1.37507
H	0.891223	1.779573	-2.12785
N	1.225296	1.687536	0.00000
H	2.166173	1.313529	0.00000
C	-0.268574	-1.708388	-1.13065
C	0.202525	-2.957775	-0.71738
C	0.202525	-2.957775	0.71738
C	-0.268574	-1.708388	1.13065
H	-0.427891	-1.294481	-2.12510
H	0.505259	-3.773586	-1.37417
H	0.505259	-3.773586	1.37417
H	-0.427891	-1.294481	2.12510
N	-0.555104	-0.972014	0.00000
H	-0.858095	-0.001968	0.00000

Supplementary Table 2: Cartesian coordinates in Å of the optimised geometry of the pyrrole dimer calculated at the MP2 level using the aug-cc-pVDZ basis set. The centres of mass of the monomers are separated by $R = 4.06$ Å, the planes of the monomers are at an angle of $\phi = 51.5°$, and the centre of mass vector has an angle of $\theta = 13.6°$ from the norm of the plane of monomer A, in good agreement with the experimentally determined values[2]: $R = 4.116$ Å, $\phi = 55.42°$, $\theta = 12.12°$.

C	1.8906435	-1.0524386	-1.1298361
C	1.9652931	-2.3893716	-0.7184910
C	1.9652931	-2.3893716	0.7184910
C	1.8906435	-1.0524386	1.1298361
H	1.8661856	-0.6076488	2.1229987
N	1.8525493	-0.2659016	0.0000000
H	1.7390034	0.7472153	0.0000000
H	2.0279883	-3.2567495	1.3754276
H	2.0279883	-3.2567495	-1.3754276
H	1.8661856	-0.6076488	-2.1229987
C	-1.8567604	-1.1111260	1.1298361
C	-3.0519030	-0.5073079	0.7184910
C	-3.0519030	-0.5073079	-0.7184910
C	-1.8567604	-1.1111260	-1.1298361
H	-1.4593321	-1.3123397	-2.1229987
N	-1.1565522	-1.4714039	0.0000000
H	-0.2223942	-1.8796288	0.0000000
H	-3.8344219	-0.1279147	-1.3754276
H	-3.8344219	-0.1279147	1.3754276
H	-1.4593321	-1.3123397	2.1229987
H	-1.5166092	1.1324135	0.0000000
N	-0.6959971	1.7373056	0.0000000
C	-0.0338832	2.1635646	1.1298361
C	1.0866100	2.8966795	0.7184910
C	1.0866100	2.8966795	-0.7184910
C	-0.0338832	2.1635646	-1.1298361
H	-0.4068535	1.9199885	-2.1229987
H	1.8064336	3.3846641	-1.3754276
H	1.8064336	3.3846641	1.3754276
H	-0.4068535	1.9199885	2.1229987

Supplementary Table 3: Cartesian coordinates in Å of the optimised geometry of the pyrrole trimer calculated at the MP2 level using the aug-cc-pVDZ basis set

State	ΔE (eV)	Dominant Configurations (Coefficients)	f
$1A''$	4.78	$\pi_B \rightarrow 3s_A$ (0.86)	0.0035
$2A''$	5.24	$\pi_A \rightarrow 3s_A$ (0.66)	0.0230
$3A''$	5.31	$\pi_A \rightarrow 3s_A$ (0.59)	0.0106
		$\pi_B \rightarrow 3p_{z,A}$ (0.56)	
$2A'$	5.32	$\pi_B \rightarrow 3p_{y,A}$ (0.85)	0.0392
$4A''$	5.53	$\pi_B \rightarrow 3d_{AB}$ (0.53)	0.0007
		$\pi_B \rightarrow \pi_A^*$ (0.41)	
		$\pi_B \rightarrow \pi_A^*$ (0.39)	
$5A''$	5.59	$\pi_B \rightarrow 3p_{z,B}$ (0.71)	0.0002
$3A'$	5.62	$\pi_B \rightarrow \pi_B^*$ (0.41)	0.0010
		$\pi_B \rightarrow 3s_A$ (0.40)	
		$\pi_B \rightarrow Ryd_?$ (0.36)	
		$\pi_B \rightarrow \pi_A^*$ (0.32)	
$4A'$	5.73	$\pi_B \rightarrow 3s_A$ (0.42)	0.0034
		$\pi_A \rightarrow \pi_A^*$ (0.41)	
		$\pi_A \rightarrow \pi_A^*$ (0.35)	
$5A'$	5.79	$\pi_B \rightarrow 3s_A$ (0.57)	0.0024
		$\pi_B \rightarrow 3p_{z,B}$ (0.33)	
$6A''$	5.88	$\pi_A \rightarrow 3p_{z,A}$ (0.62)	0.0497

		$\pi_B \rightarrow \pi^*_B$ (0.49)	
$7A''$	5.89	$\pi_A \rightarrow \pi^*_A$ (0.54)	0.1102
		$\pi_B \rightarrow 3d_{AB}$ (0.41)	
		$\pi_A \rightarrow \pi^*_A$ (0.37)	
		$\pi_A \rightarrow 3d_{AB}$ (0.32)	
$6A'$	5.89	$\pi_A \rightarrow 3p_{y,A}$ (0.71)	0.0239
$8A''$	5.96	$\pi_A \rightarrow 3p_{z,A}$ (0.60)	0.0371
		$\pi_B \rightarrow \pi^*_B$ (0.48)	
$7A'$	6.04	$\pi_B \rightarrow 3d_{AB}$ (0.62)	0.0224
		$\pi_A \rightarrow 3p_{y,A}$ (0.48)	
$9A''$	6.06	$\pi_B \rightarrow \pi^*_A$ (0.64)	0.0072
$10A''$	6.16	$\pi_B \rightarrow \pi^*_A$ (0.67)	0.0036
$8A'$	6.17	$\pi_A \rightarrow 3s_A$ (0.73)	0.0049
$9A'$	6.19	$\pi_B \rightarrow 3d_{AB}$ (0.69)	0.0055
$10A'$	6.33	$\pi_B \rightarrow 3p_{z,A}$ (0.65)	0.0131
$11A''$	6.35	$\pi_B \rightarrow 3p_{y,A}$ (0.85)	0.0007

Supplementary Table 4: Vertical excitation energies, dominant configurations and oscillator strengths of the excited states of the pyrrole dimer as calculated at the DFT-MRCI/aug-cc-pVDZ level. Subscripts of A or B appearing on the orbital labels denote which monomer the orbital is most localised on.

Supplementary Notes

Supplementary Note 1: Dimer formation. 240 nm $1 + 1$ multiphoton photoelectron spectra, recorded with the mininum and maximum He expansion pressures, are presented in Fig. 1 in the paper and were also recorded at an intermediate expansion pressure. At this wavelength, the maximum eKE that is accessible in the monomer is 2.11 eV; however, at higher expansion pressures, we see an additional feature with eKE $\approx$ 2.45 eV. The intensity of this peak in the photoelectron spectrum is linearly dependent on the intensities of the pump and probe laser pulses, which excludes multiphoton processes. The intensity of this peak increases with increasing He expansion pressure, suggesting that it arises from the formation of clusters. To monitor the formation of clusters, we recorded ion images following two-photon ionisation. The time of flight (ToF) from the interaction region to the detector depends on the mass of the ion, $t_{\mathrm{ToF}} \propto \sqrt{m}$, and we observe images of dimer ions displaced by $x_{\mathrm{py}}\sqrt{2}$ and trimer ions displaced by $x_{\mathrm{py}}\sqrt{3}$, where x_{py} is the displacement of the pyrrole monomer ions along the molecular beam axis which is parallel to the imaging detector and perpendicular to the ToF axis (Supplementary Figure 1). The integrated cluster ion counts as a percentage of monomer ion counts (10% dimer, 1% trimer) are plotted as a function of cluster size in the inset of Supplementary Figure 1 and are observed to have an approximately exponential distribution. The average cluster ion size at the detector is $\bar{N} \approx 1$. It should be noted that these measurements are lower limits for the clusters in the interaction region because they do not account for fragmentation of cluster ions on the way to the detector.

Supplementary Methods

Monomer Lifetimes: Relaxation lifetimes for the pyrrole monomer measured in a jet using 0.4 bar He carrier gas with excitation at 249.5 nm (4.97 eV), 245 nm (5.06 eV), 240 nm (5.17 eV) and 200 nm (6.20 eV) and a probe wavelength of 300 nm (4.13 eV) are listed in Supplementary Table 1.

Ab initio calculations: Vertical excitation energies and vertical ionisation energies in the paper are

reported for the pyrrole dimer and trimer optimised at the MP2-SCS level using the aug-cc-pVDZ basis set and the Turbomole program. The optimised coordinates are listed below in Supplementary Tables 2 and 3. The vertical excitation energies and dominant configurations for the lowest 20 states of the pyrrole dimer calculated at the MRCI/DFT level using the same basis set are listed in Supplementary Table 4

Supplementary References

[1] Wu, G. *et al.* Excited state non-adiabatic dynamics of pyrrole: A time-resolved photoelectron spectroscopy and quantum dynamics study. *J. Chem. Phys.* **142**, 074302 (2015).

[2] Columberg, G. & Bauder, A. Pure rotational spectrum, quadrupole coupling constants and structure of the dimer of pyrrole. *J. Chem. Phys.* **106**, 504–510 (1997).
